# Effect of exercise on cognitive function and synaptic plasticity in Alzheimer's disease models: A systematic review and meta-analysis

**DOI:** 10.3389/fnagi.2022.1077732

**Published:** 2023-01-10

**Authors:** Linlin Guo, Xinxin Yang, Yuanyuan Zhang, Xinyi Xu, Yan Li

**Affiliations:** ^1^College of Nursing, Hebei Medical University, Shijiazhuang, China; ^2^Postdoctoral Research Station in Basic Medicine, Hebei Medical University, Shijiazhuang, China; ^3^Neuroscience Research Center, Hebei Medical University, Shijiazhuang, China; ^4^Hebei Key Laboratory of Neurodegenerative Disease Mechanism, Shijiazhuang, China

**Keywords:** exercise, cognitive function, synaptic plasticity, Alzheimer's disease, animal

## Abstract

**Introduction:**

Cognitive decline is a central manifestation of Alzheimer's disease (AD), and its process is inseparable from changes in synaptic plasticity. The aim of this review was to summarize and evaluate the effectiveness of exercise on cognitive function and synaptic plasticity in AD animal models.

**Materials and methods:**

Eligible studies were searched from PubMed, MEDLINE, EMBASE, Web of Science, and Cochrane Library from April to May 2022. The risk of bias was evaluated by Systematic Review Centre for Laboratory Animal Experimentation (SYRCLE). The Morris water maze (MWM) test and synaptic plasticity were considered outcome measures. Data were analyzed using random-effects meta-analyses using the software Stata. Heterogeneity was examined by using I2 test. Sensitivity analysis and publication bias were also assessed.

**Results:**

A total of 20 randomized controlled studies were eligible for study inclusion. Compared with controls, exercise decreased escape latency (SMD = −0.86, 95% CI: −1.21 to −0.50, *P* < 0.001), increased platform crossover numbers (SMD = 1.34, 95% CI: 0.57–2.11, *P* = 0.001) and time in the target quadrant (SMD = 1.65, 95% CI: 0.95–2.36, *P* < 0.001) and the expression of PSD95 (SMD = 0.73, 95% CI: 0.25–1.21, *P* = 0.003) in AD animals. The results of the subgroup analysis showed that exercise before AD had a greater effect on escape latency (SMD = −0.88, 95% CI: −1.25 to −0.52, *P* < 0.001), platform crossover numbers (SMD = 1.71, 95% CI: 1.23–2.18, *P* < 0.001), time in the target quadrant (SMD = 2.03, 95% CI: 1.19–2.87, *P* < 0.001) and the expression of PSD95 (SMD = 0.94, 95% CI: 0.19–1.69, *P* = 0.014) than exercise after AD. The results of the subgroup analysis also showed that treadmill running might be an appropriate exercise type.

**Conclusion:**

Our findings suggested that exercise had a potential effect on improving cognitive function and synaptic plasticity. It can play a better neuroprotective role before AD.

**Systematic review registration:**

PROSPERO, identifier: CRD42022328438.

## 1. Introduction

Alzheimer's disease (AD) is a fatal, aging-related, and gradually progressing brain condition that is characterized by memory loss and cognitive deterioration (Alzheimer's disease facts and figures, 2022). AD is the most prevalent form of dementia that may contribute to 60–70% of cases (Soria et al., 2019), and it places a heavy financial burden on society (Tahami Monfared et al., 2022). In approximately the next three decades, this societal burden, which is currently estimated to be worth over $958 billion globally, is projected to multiply many times (Jia et al., 2018; Cimler et al., 2019; Clay et al., 2019). It is rapidly turning into one of the most costly, fatal, and serious diseases of this century (Livingston et al., 2020). The World Health Organization (WHO) has recognized AD as a global public health priority (WHO, 2021). It is predicted that 12.7 million persons aged 65 and older will have AD by 2050 (Alzheimer's disease facts and figures, 2022). The number and percentage of older persons with AD will increase together with the expansion of the population 65 and older. In summary, AD is emerging as a growing and pervasive threat to global health.

The pathology of AD is characterized by the accumulation of amyloid-β (Aβ) plaques and tau neurofibrillary tangles in the hippocampus, both of which are essential for learning and memory (Busche and Hyman, 2020). Previous studies have shown that Aβ and tau can harm memory by preventing synaptic plasticity in the hippocampus (Forner et al., 2017). This synaptic plasticity includes activity-dependent changes in synaptic remodeling, axonal sprouting, dendritic remodeling, dendritic spine dynamics, and synaptic proteins. In a concentration-dependent manner, oligomer Aβ can impair long-term potentiation (LTP) while enhancing long-term depression (LTD), the consequent disruption of glutamatergic transmission results in the loss of dendritic spines and causes memory deficits (Rajmohan and Reddy, 2017). Before plaque development, synaptic function damage was seen in young mice, which suggested that Aβ oligomers caused synaptic abnormalities beforehand (Hong et al., 2016). Synaptic pathology is thus an early stage of disease development. However, currently, there is no treatment available to target synaptic damage. In addition, the effectiveness of current disease-modifying therapy and anti-dementia medications is limited (Lane et al., 2018). Therefore, it is urgent to seek alternative therapies to prevent and treat synaptic damage.

Excitingly, there have been multiple randomized controlled trials (RCTs) and several reviews provided support for the beneficial effects of exercise on cognitive function and synaptic plasticity of AD (Choi et al., 2018; Da Costa Daniele et al., 2020; De Miguel et al., 2021). Both the WHO (2021) and the NICE guidelines (NICE, 2015) recommend implementing exercise in the standard treatment of AD. Exercise may exert beneficial effects on cognitive function and synaptic plasticity through various mechanisms. Such as promoting the release of neurogenic factors (Choi et al., 2018), maturation of new neurons (Lattanzi et al., 2022), and cerebral angiogenesis (Tang et al., 2018), which ultimately increases neurogenesis and upregulates synaptic protein expression. Postsynaptic proteins were reported to be lost at a higher rate than presynaptic proteins in AD (Gylys et al., 2004). Postsynaptic density protein 95 (PSD95), the most important and abundant scaffolding protein of the postsynaptic membrane, is mainly found in the mature excitatory glutamatergic synapse (Coley and Gao, 2018; Mardones et al., 2019). It is necessary for receptor activity and stability of the postsynaptic membrane (Dore et al., 2021), which could be affected by exercise. However, controversial results about the effects of exercise on cognition and synapses have also been reported (Wang et al., 2021). Meanwhile, there is no systematic review of the effects of exercise on synaptic plasticity to date. Therefore, it is thus necessary to update the knowledge about the effects of exercise on cognitive function and synaptic plasticity.

Thus, based on the current controversies and the limitations of the systematic reviews, the purpose of this study is to systematically review the current literature that evaluated the effect of exercise on AD model in MWM tests and synaptic plasticity to determine: (i) the positive effects of exercise on cognitive function and synaptic plasticity; (ii) the differential preventive and therapeutic effects of exercise, and (iii) the different effects of exercise types on cognitive function and synaptic plasticity.

## 2. Materials and methods

This systematic review was designed following the writing guidelines in Preferred Reporting Items for Systematic Reviews and Meta-Analyses (PRISMA) 2020 explanation and elaboration: updated guidance and exemplars for reporting systematic reviews (Page et al., 2021). The study was registered in The International Prospective Register of Systematic Reviews (PROSPERO), and the number was CRD42022328438. All of the studies retrieved were animal studies related to exercise and cognitive function.

### 2.1. Search strategy

PubMed, MEDLINE, EMBASE, Web of Science, and Cochrane Library were searched for studies that were published between April 1, 2012 and April 1, 2022. The reference lists of previous systematic reviews were carefully examined for new references. The strategy was formulated based on the combination of the Medical Subject Headings (MeSH) and free text terms as follows: (exercise OR resistance training OR physical exercise OR aerobic OR treadmill OR running OR voluntary OR involuntary OR swimming) AND (synapse^*^ OR neuronal plasticity OR synaptic OR neuroplasticity OR plasticity OR synaptogenesis OR dendritic OR dendron OR long-term potentiation OR LTP) AND (Alzheimer disease OR dementia OR Alzheimer OR AD OR cognition). The detailed search strategy was provided in the Supplementary material.

### 2.2. Inclusion and exclusion criteria

#### 2.2.1. Inclusion criteria

Studies were considered if all of the following standards were met: (1) had animal models for Alzheimer's disease, either by genetic variants (transgenic) or drug-induced; (2) included both control group (sedentary) and exercise group; (3) had Morris Water Maze (MWM) test or at least one indicator of synaptic plasticity; (4) randomized controlled trials; (5) published in English.

#### 2.2.2. Exclusion criteria

Studies were excluded if any of the following standards were met: (1) using physical exercise combined with any type of medicines or other non-pharmacological treatments in the animal model; (2) absence of full text, literature review studies, course completion papers, dissertations, theses, and annals abstracts.

### 2.3. Data extraction

Two reviewers collected data based on the following lists separately. The divergences were solved by consulting a third reviewer.

Extracted data from each study included: (1) the first author's name, publication year; (2) animal data including the species, gender, age, and sample size of each group; (3) exercise protocol including type, intensity, time, frequency, and duration of exercise; (4) outcome assessment including escape latency, number of platform crossings, time in the target quadrant selected in the MWM test and the biomarkers of synaptic plasticity. The final result was extracted if outcomes were presented at different time points; (5) the analyzed brain region; (6) the timing of the implementation of exercise: according to previous reports, the pathological changes (diffuse amyloid plaques) were first observable in the brain for particular mouse model of 5^*^ FAD (Gatt et al., 2019), APP/PS1 (Zhu et al., 2017) and 3^*^ Tg (Javonillo et al., 2022) at 1.5–2 months of age, 3 months of age, and 4 months of age, respectively. According to the characteristics of each animal model, we divided the animals included into exercise before AD group and exercise after AD group.

The data such as mean and standard deviation (SD) or standard error of mean (SEM), which were not provided in the texts or tables, were extracted from the graphs through the free software WebPlotDigitizer (Drevon et al., 2017). By multiplying the reported SEM by the square root of the sample size to convert to SD we used (Vasconcelos-Filho et al., 2021).

### 2.4. Quality assessment

Two reviewers assessed study quality independently using a ten-item scale introduced by “SYRCLE's Risk of Bias (RoB) tool for animal studies” (Hooijmans et al., 2014), and the divergences were solved by consulting a third reviewer. The criteria for evaluation under the tool were: random allocation sequence; similar baseline characteristics; allocation concealment; random housing; blinded intervention; random selection for outcome assessment; blinded assessment of outcome; incomplete outcome data; selective outcome reporting; other sources of bias. Each criterion in a 10-point scale for a quality index was assigned a point value of one. A “Yes” response indicated a low risk of bias, a “No” response indicated a high risk of bias, and a “NC” response indicated a level of prejudice that was unsure due to insufficient data. Each item received one point for answering “Yes”.

### 2.5. Statistical analysis

Meta-analysis was performed using the software Stata (Version 17.0). The mean ± SD was calculated with 95% confidence interval (CI) and standardized mean difference (SMD) using randomized effect models to account for potential heterogeneity. I2 test was used to calculate the degree of heterogeneity among studies, with values 30%, 30–60%, and >60% representing low, moderate, and high levels of heterogeneity, respectively (Hernandez et al., 2020). When there was high heterogeneity, sensitivity analysis was performed by eliminating the data one by one to examine its impact on the result. Funnel plots and Egger's test were used to evaluate publication bias.

## 3. Results

**Figure 1:**
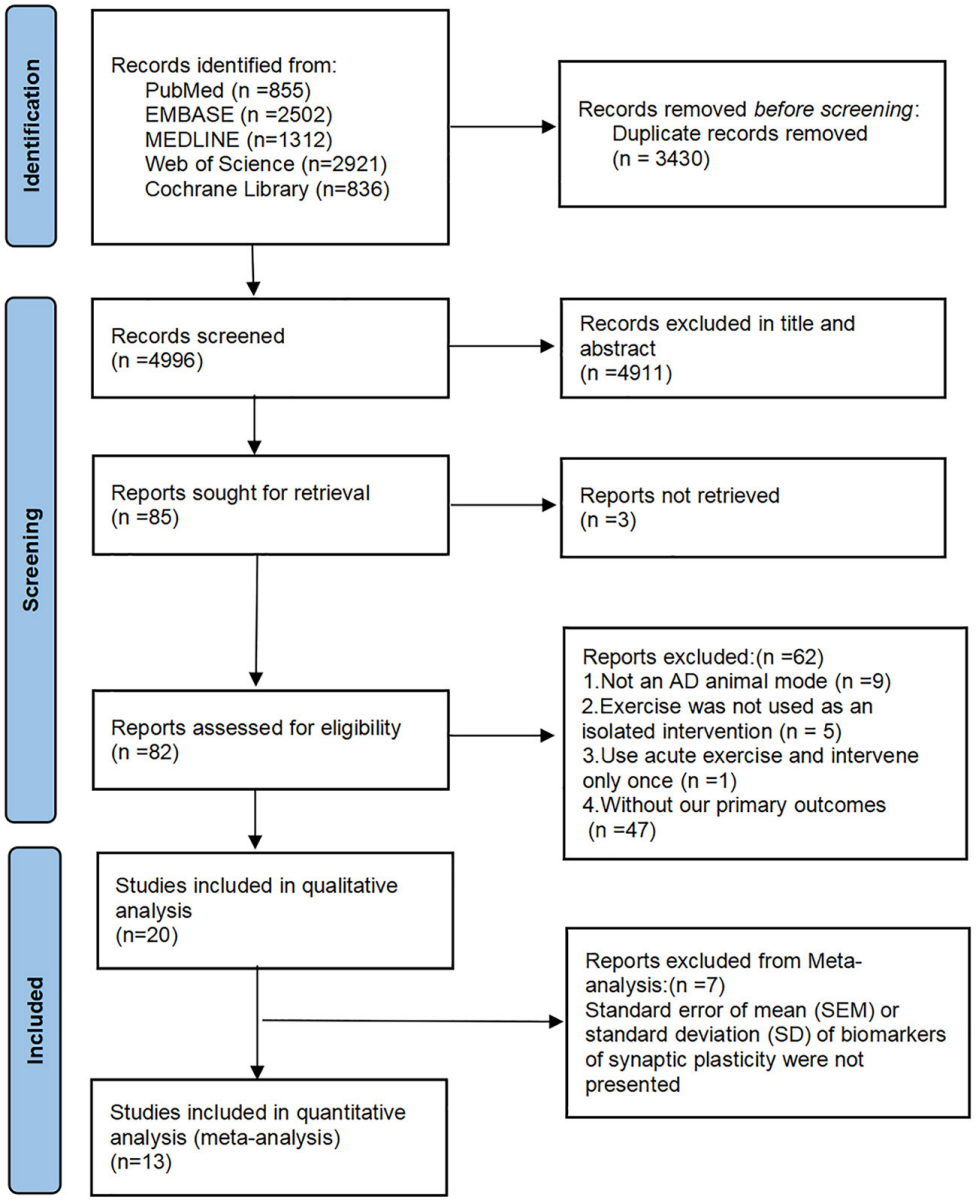
The flow diagram of the study selection process.

A total of 8,426 studies were found in the initial search, and after the removal of duplicates, 4,996 remained for screening. Of these, 85 full-text articles were reviewed after screening titles and abstracts. After reviewing the full texts, 62 studies were excluded for the following reasons: nine for animals that were not models of AD; five for no exercise as an isolated intervention; one for no long-term exercise; 47 for no assessments or reports of results related to MWM or synaptic plasticity. In the end, 20 studies met the inclusion criteria and were included in the qualitative analysis (Wang et al., 2013, 2021; Kim et al., 2014; Revilla et al., 2014; Cho et al., 2015; Dao et al., 2015, 2016; Zhao et al., 2015, 2020; Wu et al., 2018; Lourenco et al., 2019; Belaya et al., 2020; Liu et al., 2020, 2022; Li et al., 2021, 2022; Mu et al., 2022; Park et al., 2022; Xu et al., 2022; Yang et al., 2022). Twelve for the quantitative analysis because of the lacked data on synaptic plasticity of the eight articles (Revilla et al., 2014; Cho et al., 2015; Belaya et al., 2020; Liu et al., 2020, 2022; Zhao et al., 2020; Li et al., 2021, 2022; Wang et al., 2021; Mu et al., 2022; Park et al., 2022; Xu et al., 2022). The flow diagram of the selection process is shown in Figure 1.

### 3.1. Study characteristics

The included studies used two species: mice (*n* = 15) and rats (*n* = 5). AD models were drug-induced and transgenic, with the latter being more prevalent. Transgenic models included: APP/PS1 mice (*n* = 6), 3×Tg-AD mice (*n* = 6), 5×FAD mice (*n* = 1), TgF344 rats (*n* = 1). The non-transgenic models included: injection of Aβ (*n* = 5) and streptozotocin (*n* = 1). There were 15 studies only using male animals, one using both sexes, and four of them did not describe the sex of animals used in the experiment. Sixteen studies mentioned age of the experimental animals. In mouse-related studies, age ranged from 1 to 24 months; in rat-related studies, age ranged from 2 to 2.5 months.

Four types of intervention were used in the included studies: treadmill exercise (*n* = 10), wheel running (*n* = 7), swimming (*n* = 2) and resistance training (*n* = 1). The duration of the intervention ranged from 1 to 8 months. However, only one study was conducted for 12 days. Behavioral tests were carried out in 16 studies to assess animal learning and memory function: MWM (*n* = 10), Novel Object Recognition (NOR, *n* = 5), and Y-maze test (*n* = 3). Several relevant indicators for evaluating synaptic plasticity were applied, for example, dendritic spine density through Golgi staining, the number of synapses through electron microscopy, LTP through *in vivo* electrophysiology, and several synaptic-associated proteins through western blotting. PSD95 was analyzed in the hippocampus (*n* = 11) and cortex (*n* = 4), and one of the studies analyzed the length of the apical dendrites. It should be noted that a total of 20 studies were included in this review. Since four studies used two groups of RCTs, respectively, the characteristics of 24 RCTs need to be described. All these data can be found in Table 1.

**Table 1:** Characteristics of the included studies.

**Study**	**AD model**	**Exercise protocol**	**Start exercise time**	**Outcome measures**	**Main molecular results**
Yang et al. (2022)	TgF344 rats (*n* = 11), male, 2-month	Treadmill running:18 m/min, 45 min/day, 3 days/week Duration: 8 months	Before AD	Dendritic spine density: Golgi staining; the number of synapses: electron microscopy	None
Xu et al. (2022)	3×Tg mice (*n* = 11), male, 8-week	Wheel running: 1 h/day, 5 days/week Duration: 5 months	Before AD	Cognition: MWM, NOR; dendritic spine density: Golgi staining; PSD95, SYN: IHC,WB	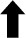 Hippocampus (PSD95, SYN)
Mu et al. (2022)	3×Tg mice (*n* = 10), male, 3-month	Treadmill running:12 m/min^*^10 min +15 m/min^*^50 min, 1 h/day, 5 days/week Duration: 12 weeks	Before AD	Dendritic spine density: Golgi staining; the number of synapses: electron microscopy; PSD95, SYN: WB	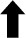 Hippocampus and cortex (PSD95, SYN)
Li et al. (2022)	Aβ induced ICR mice, male, 8-week	Wheel running: 24 h/day Treadmill exercise: 5 m/min^*^5 min + 10 m/min^*^40 min+3 m/min^*^5 min, 50 min/day, 6 days/week Duration: 4 weeks	After AD	Cognition: MWM, NOR; PSD95, SYP: WB	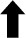 Hippocampus (PSD95, SYP)
Liu et al. (2020)	APP/PS1 mice (*n* = 12), male, 3-month	Wheel running: 4 h/day, 5 days/week Duration: 8 weeks	Before AD	Cognition: MWM, NOR, Y-maze test; PSD95, SYN: WB	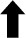 Hippocampus (PSD95, SYN)
Liu et al. (2022)	APP/PS1 mice (*n* = 12), male, 7-month	Wheel running: 4 h/day, 5 days/week Duration: 8 weeks	After AD	Cognition: MWM, NOR, Y-maze test; PSD95, SYN: WB	None
Park et al. (2022)	3×Tg mice (*n* = 10), male, 5-month	Treadmill running: 10 m/min^*^40 min (1–3 w) + 11 m/min^*^40 min (4–6 w) + 12 m/min^*^50 min (7–9 w) + 13 m/min^*^50 min (10–12 w), 6 days/week Duration: 8 weeks	After AD	Cognition: MWM; PSD95, SYN: WB	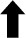 Hippocampus (PSD95, SYN)
Wang et al. (2021)	APP/PS1 mice (*n* = 7), male, 10-week	Wheel running Duration: 16 weeks	Before AD	Cognition: MWM; PSD95, SYN: WB	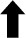 Hippocampus and cortex (SYN)
Wang et al. (2021)	APP/PS1 mice (*n* = 7), male, 24-week	Wheel running Duration: 16 weeks	After AD	Cognition: MWM; PSD95, SYN: WB	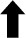 Hippocampus and cortex (SYN)
Li et al. (2021)	APP/PS1 mice (*n* = 15), 3-month	Treadmill running: 5 m/min^*^5 min + 8 m/min^*^5 min + 12 m/min^*^30 min + 5 m/min^*^5 min, 45 min/day, 5 days/week Duration:12 weeks	Before AD	Cognition: MWM; the number of synapses: electron microscopy. SYN, PSD95, MAP2,: WB	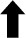 Hippocampus (SYN, PSD95, MAP2)
Belaya et al. (2020)	5×FAD mice (*n* = 20), male, 6-week	Wheel running Duration:6 months	Before AD	Cognition: MWM; PSD95, SYN: WB	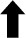 Hippocampus (PSD95)
Liu et al. (2020)	3×Tg mice (*n* = 8), male, 9-month	Resistance training: weight-bearing climbing, once every 2 days Duration:4 weeks	After AD	Cognition: NOR, Y-maze test; PSD95: WB	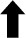 Hippocampus and cortex (PSD95)
Zhao et al. (2020)	APP/PS1 mice (*n* = 9), male, 3-month	Treadmill running: 5 m/min^*^5 min + 8 m/min^*^5 min + 12 m/min^*^30 min + 5 m/min^*^5 min, 45 min/day, 5 days/week Duration:12 weeks	Before AD	Cognition: MWM; SYN, GAP43: WB	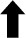 Hippocampus (SYN, GAP43)
Lourenco et al. (2019)	APP/PS1 mice, male and female, 2.5–3-month	Swimming: 20 min/d, 5 days/week Duration:3 weeks	Before AD	LTP: vivo electrophysiology;	None
Wu et al. (2018)	Streptozotocin-induced Sprague–Dawley rats (*n* = 9), male, 2.5-month	Swimming: began at 10 min/day and was increased by 10 min every 2 d until the duration reached 1 h/day, 5 days/week Duration:4 weeks	Before AD	Cognition: NOR; SYN: confocal microscopy	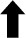 Hippocampus (SYN)
Dao et al. (2016)	Aβ-induced Wistar rats, male	Treadmill running: 10 m/min^*^30 min (1–2 w)+15 m/min^*^45 min (3–4 w), 5 days/week Duration:4 weeks	After AD	LTP: vivo electrophysiology	None
Dao et al. (2015)	Aβ-induced Wistar rats, male	Treadmill running: 10 m/min^*^30 min (1–2 w) + 15 m/min^*^45 min (3–4 w), 5days/week Duration:4 weeks	Not clear	LTP: vivo electrophysiology	None
Cho et al. (2015)	3×Tg mice (*n* = 12), 4-month	Treadmill running: 5 m/min^*^5 min + 10 m/min^*^20 min + 5 m/min^*^5 min, 30 min/day, 5 days/week Duration:12 weeks	Before AD	Cognition: MWM; PSD95, SYN:WB	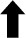 Hippocampus and cortex (PSD95, SYN)
Cho et al. (2015)	3×Tg mice (*n* = 12), 24-month	Treadmill running:5m/min^*^5min +10m/min^*^20min+5m/min^*^5min, 30min/day, 5days/week Duration:12 weeks	After AD	Cognition: MWM; PSD95, SYN:WB	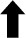 Hippocampus and cortex (PSD95, SYN)
Zhao et al. (2015)	APP/PS1 mice (*n* = 12), 3-month	Treadmill running: 5 m/min^*^5 min + 8 m/min^*^5 min + 11 m/min^*^20 min, 30 min/day, 5 days/week Duration: 5 months	Before AD	Cognition: MWM; LTP: vivo electrophysiology	None
Zhao et al. (2015)	APP/PS1 mice (*n* = 12), 12-month	Treadmill running: 5 m/min^*^5 min + 8 m/min^*^5 min + 11 m/min^*^20 min, 30 min/day, 5 days/week Duration:5 months	After AD	Cognition: MWM; LTP: vivo electrophysiology	None
Revilla et al. (2014)	3×Tg mice, 1-month	Wheel running Duration:6 months	Before AD	PSD95, SYN: WB	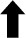 Hippocampus (PSD95, SYN)
Kim et al. (2014)	Aβ-induced Sprague-Dawley rats (*n* = 10), male, 7-week	Treadmill running: 3 m/min^*^5 min + 5 m/min^*^5 min + 8 m/min^*^20 min, 30 min/day, 5 days/week Duration:4 weeks	After AD	Apical dendritic length: electron microscopy	None
Wang et al. (2013)	Aβ-induced C57bl/6 mice, male, 2-month	Wheel running Duration: 12 days	After AD	Cognition: Y-maze test; SYN: IHC	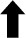 Hippocampus (SYN)

MWM, Morris water maze; NOR, Novel Object Recognition; LTP, long-term potentiation; PSD95, Postsynaptic density protein 95; SYN, Synaptophysin; SYP, Synaptophysin; MAP2, Microtubule-associated protein-2; GAP43, Growth associated protein-43; IHC, Immunohistochemistry; WB, Western Blotting.

### 3.2. Study quality evaluation

Table 2 showed the methodological quality assessments of the 20 included studies, with study quality scores ranging from 3 to 6 out of a total 10. No study was considered to have low risk of sequence generation and allocation concealment (selection bias), blinding (performance bias) and random outcome assessment (detection bias). Fourteen studies were thought to have low risk of baseline characteristics. Seventeen studies were judged as having low risk of random housing. Sixteen studies were thought to have low risk of incomplete data. However, only four studies were thought to have low risk of blinding against detection bias. All 20 studies were considered with low risk for selective outcome reporting and other sources of bias.

**Table 2:** The methodological quality assessments of 20 included studies.

**Study**	**A**	**B**	**C**	**D**	**E**	**F**	**G**	**H**	**I**	**J**	**Score**
Yang et al. (2022)	NC	Y	NC	Y	NC	NC	NC	Y	Y	Y	5
Xu et al. (2022)	NC	Y	NC	NC	NC	NC	Y	Y	Y	Y	5
Mu et al. (2022)	NC	Y	NC	Y	NC	NC	NC	Y	Y	Y	5
Li et al. (2022)	NC	Y	NC	Y	NC	NC	NC	Y	Y	Y	5
Liu et al. (2022)	NC	Y	NC	Y	NC	NC	Y	Y	Y	Y	6
Park et al. (2022)	NC	Y	NC	Y	NC	NC	NC	Y	Y	Y	5
Wang et al. (2021)	NC	Y	NC	Y	NC	NC	NC	Y	Y	Y	5
Li et al. (2021)	NC	N	NC	Y	NC	NC	Y	Y	Y	Y	5
Belaya et al. (2020)	NC	Y	NC	Y	NC	NC	NC	Y	Y	Y	5
Liu et al. (2020)	NC	Y	NC	Y	NC	NC	NC	NC	Y	Y	4
Zhao et al. (2020)	NC	Y	NC	Y	NC	NC	NC	NC	Y	Y	4
Lourenco et al. (2019)	NC	N	NC	Y	NC	NC	NC	NC	Y	Y	3
Wu et al. (2018)	NC	Y	NC	NC	NC	NC	NC	Y	Y	Y	4
Dao et al. (2016)	NC	Y	NC	Y	NC	NC	Y	Y	Y	Y	6
Dao et al. (2015)	NC	NC	NC	Y	NC	NC	NC	Y	Y	Y	4
Cho et al. (2015)	NC	NC	NC	NC	NC	NC	NC	Y	Y	Y	3
Zhao et al. (2015)	NC	NC	NC	Y	NC	NC	NC	Y	Y	Y	4
Revilla et al. (2014)	NC	NC	NC	Y	NC	NC	NC	Y	Y	Y	4
Kim et al. (2014)	NC	Y	NC	Y	NC	NC	NC	NC	Y	Y	4
Wang et al. (2013)	NC	Y	NC	Y	NC	NC	NC	Y	Y	Y	5

A—random allocation sequence; B—similar baseline characteristics; C—allocation concealment; D—random housing; E—blinded intervention; F—random selection for outcome assessment; G—blinded assessment of outcome; H—incomplete outcome data; I—selective outcome reporting; J—other sources of bias. Y, yes; N, no; NC, unclear.

### 3.3. Results of the meta-analysis

#### 3.3.1. Effect of exercise on cognitive function in AD models: Escape latency of MWM

**Figure 2:**
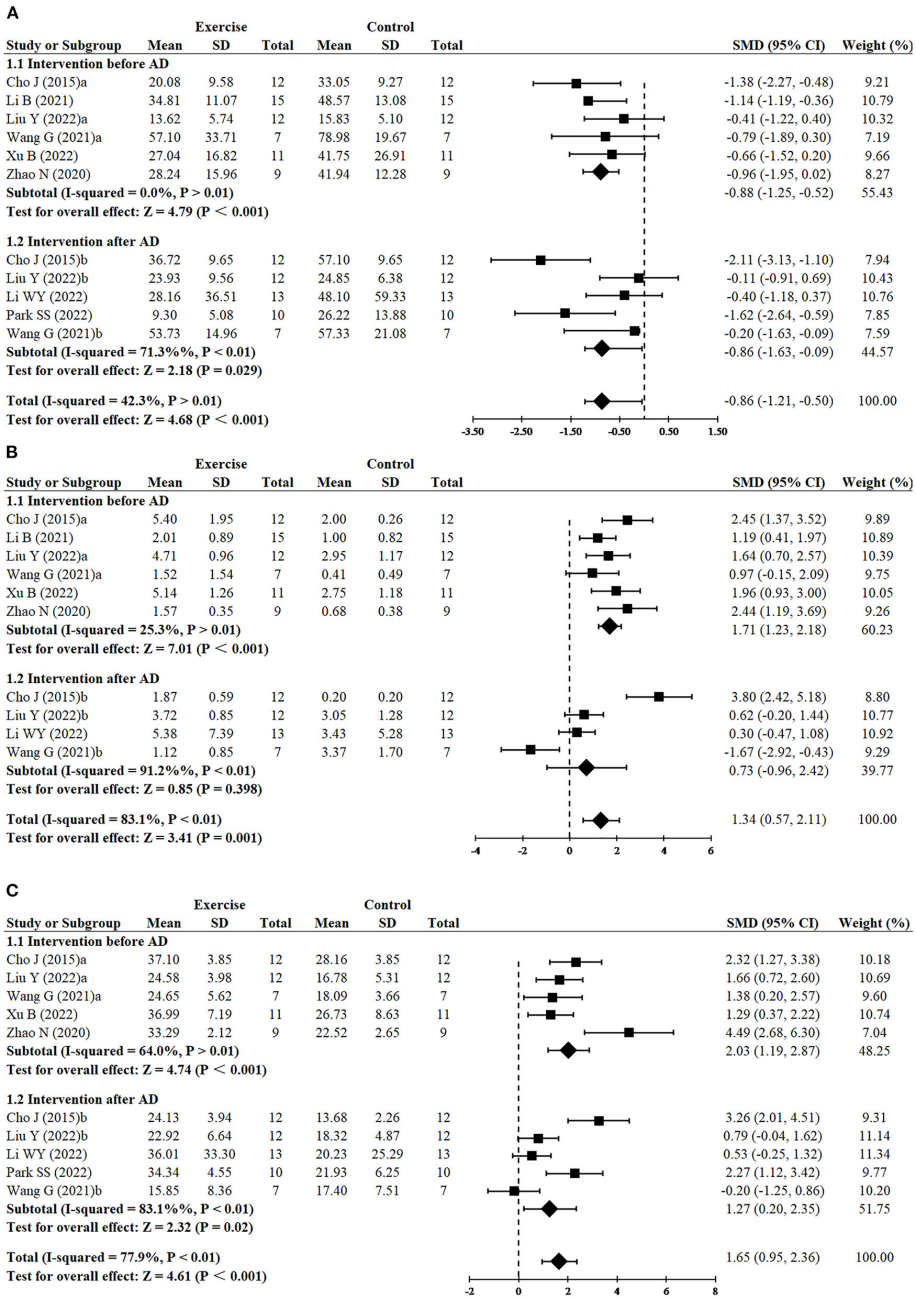
Forest plots showing the effect of exercise on cognitive function in AD models. **(A)** The effect of exercise on the escape latency of MWM. **(B)** The effect of exercise on the number of platform crossings of MWM. **(C)** The effect of exercise on the time in the target quadrant of MWM.

Since three studies (Cho et al., 2015; Wang et al., 2021; Liu et al., 2022) performed RCTs for exercise intervention before and after AD, finally, 11 RCTs from eight studies (Cho et al., 2015; Zhao et al., 2020; Li et al., 2021, 2022; Wang et al., 2021; Liu et al., 2022; Park et al., 2022; Xu et al., 2022) including 240 animals reported the impact of exercise on decreasing escape latency compared with the control group. The results demonstrated that the exercise group had a significant effect on decreasing escape latency compared with the control group (SMD = −0.86, 95% CI: −1.21 to −0.50, *P* < 0.001). There was a moderate heterogeneity between the studies (I^2^ = 42.3%). The results of the subgroup analysis showed that exercise had significant effects in both the before AD group (*n* = 6, SMD = −0.88, 95% CI: −1.25 to −0.52, *P* < 0.001) and the after AD group (*n* = 5, SMD = −0.86, 95% CI: −1.63 to −0.09, *P* = 0.029) (Figure 2A).

#### 3.3.2. Effect of exercise on cognitive function in AD models: Number of platform crossings of MWM

Since three studies (Cho et al., 2015; Wang et al., 2021; Liu et al., 2022) performed RCTs for exercise intervention before and after AD, finally, number of platform crossings was adopted as an outcome in 10 RCTs from seven studies (Cho et al., 2015; Zhao et al., 2020; Li et al., 2021, 2022; Wang et al., 2021; Liu et al., 2022; Xu et al., 2022) including 220 animals. All these studies except for one study (Wang et al., 2021) reported the positive effect of exercise on increasing platform crossover numbers (SMD = 1.34, 95% CI: 0.57–2.11, *P* = 0.001). There was a high heterogeneity between the studies (I^2^ = 83.1%). In the subgroup analysis of platform crossover numbers, there was a significant effect of exercise was observed in the before AD group (*n* = 6, SMD = 1.71, 95% CI: 1.23–2.18, *P* < 0.001), but no significant difference was found in the after AD group (*n* = 4, SMD = 0.73, 95% CI: −0.96 to 2.42, *P* = 0.398). It instructed that exercise after AD didn't increase platform crossover numbers (Figure 2B). The remaining study did not mention the relevant data and failed for meta-analysis.

#### 3.3.3. Effect of exercise on cognitive function in AD models: Time in the target quadrant of MWM

Since three studies (Cho et al., 2015; Wang et al., 2021; Liu et al., 2022) performed RCTs for exercise intervention before and after AD, finally, 10 RCTs from seven studies (Cho et al., 2015; Zhao et al., 2020; Wang et al., 2021; Li et al., 2022; Liu et al., 2022; Park et al., 2022; Xu et al., 2022) including 210 animals adopted time in the target quadrant as an outcome indicator. The results of the meta-analysis displayed that the exercise group had a significant effect on increasing time in the target quadrant, compared with the control group (SMD = 1.65, 95% CI: 0.95–2.36, *P* < 0. 001). There was a high heterogeneity between the studies (I^2^ = 77.9%). The results of the subgroup analysis showed that exercise had significant effects in both the before AD group (*n* = 5, *P* < 0.001) and the after AD group (*n* = 5, *P* = 0.02), however, compared with exercising after AD, exercising before AD seemed to have a more striking effect on increasing time in the target quadrant (SMD = 2.03, 95% CI: 1.19–2.87 vs. SMD = 1.27, 95% CI: 0.20–2.35) (Figure 2C). Likewise, the remaining study did not mention the data of the time in the target quadrant and failed for meta-analysis.

#### 3.3.4. Effect of exercise on synaptic plasticity in AD models

**Figure 3:**
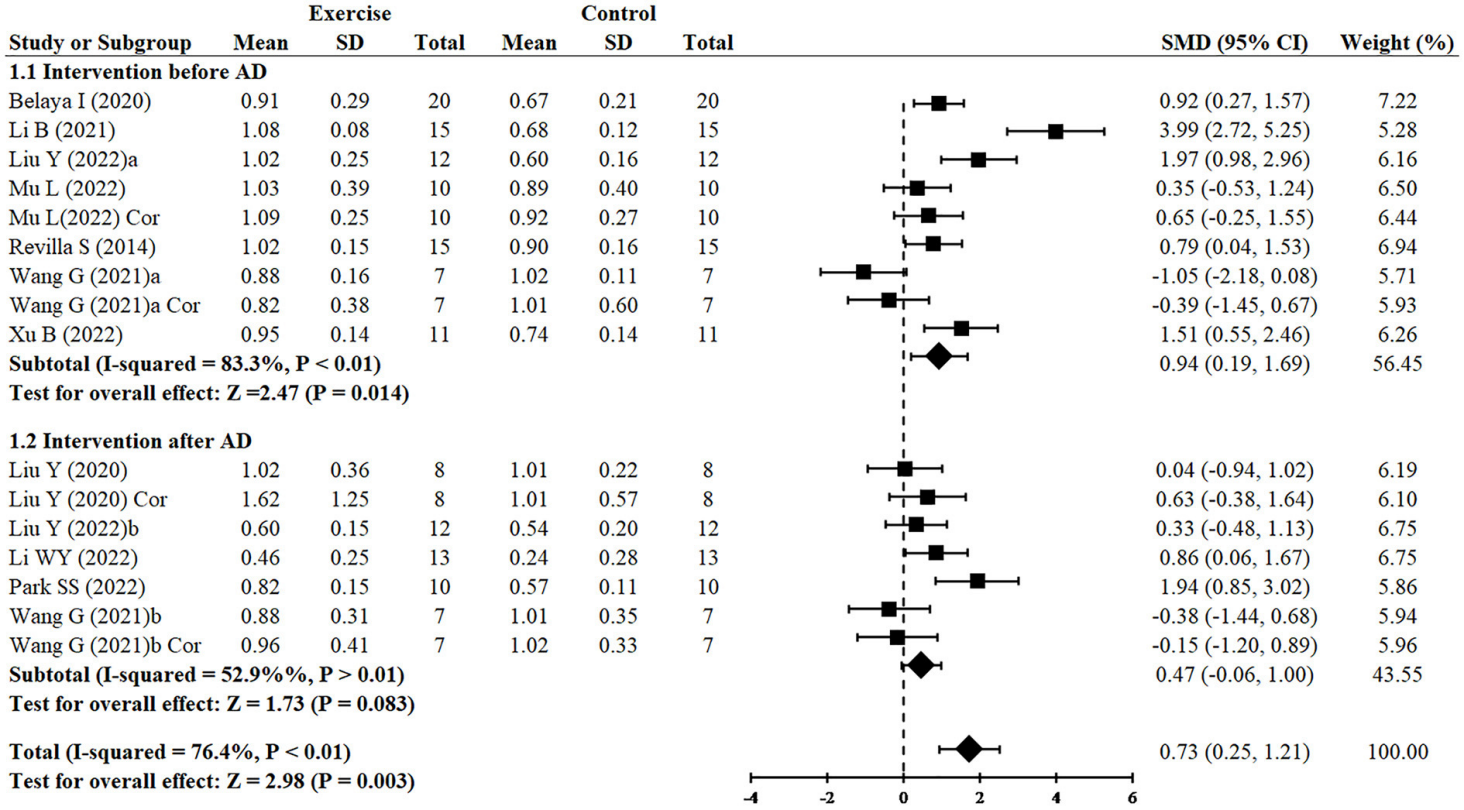
Forest plot showing the effect of exercise on the expression of PSD95 in AD models.

Since two studies (Wang et al., 2021; Liu et al., 2022) performed RCTs for exercise intervention before and after AD and three analyzed both hippocampus and cortex (Liu et al., 2020; Wang et al., 2021; Mu et al., 2022), finally, 16 RCTs from 10 studies (Revilla et al., 2014; Belaya et al., 2020; Liu et al., 2020, 2022; Li et al., 2021, 2022; Wang et al., 2021; Mu et al., 2022; Park et al., 2022; Xu et al., 2022) including 344 animals reported the effect of exercise on PSD95 expression of animals with AD. All of them except for one study (Wang et al., 2021) provided detailed data to show the significant effects of exercise on increasing the expression of PSD95 compared with the control group (SMD = 0.73, 95% CI: 0.25–1.21, *P* = 0.003). There was a high heterogeneity between the studies (I^2^ = 76.4%). In the subgroup analysis of PSD95 expression, exercise before AD group showed a significant effect (*n* = 7, SMD = 0.94, 95% CI: 0.19–1.69, *P* = 0.014), whereas exercise after AD group had no significant difference (*n* = 5, SMD = 0.47, 95% CI: −0.06 to 1.00, *P* = 0.083) (Figure 3).

#### 3.3.5. Effect of exercise type on cognitive function in AD models

**Figure 4:**
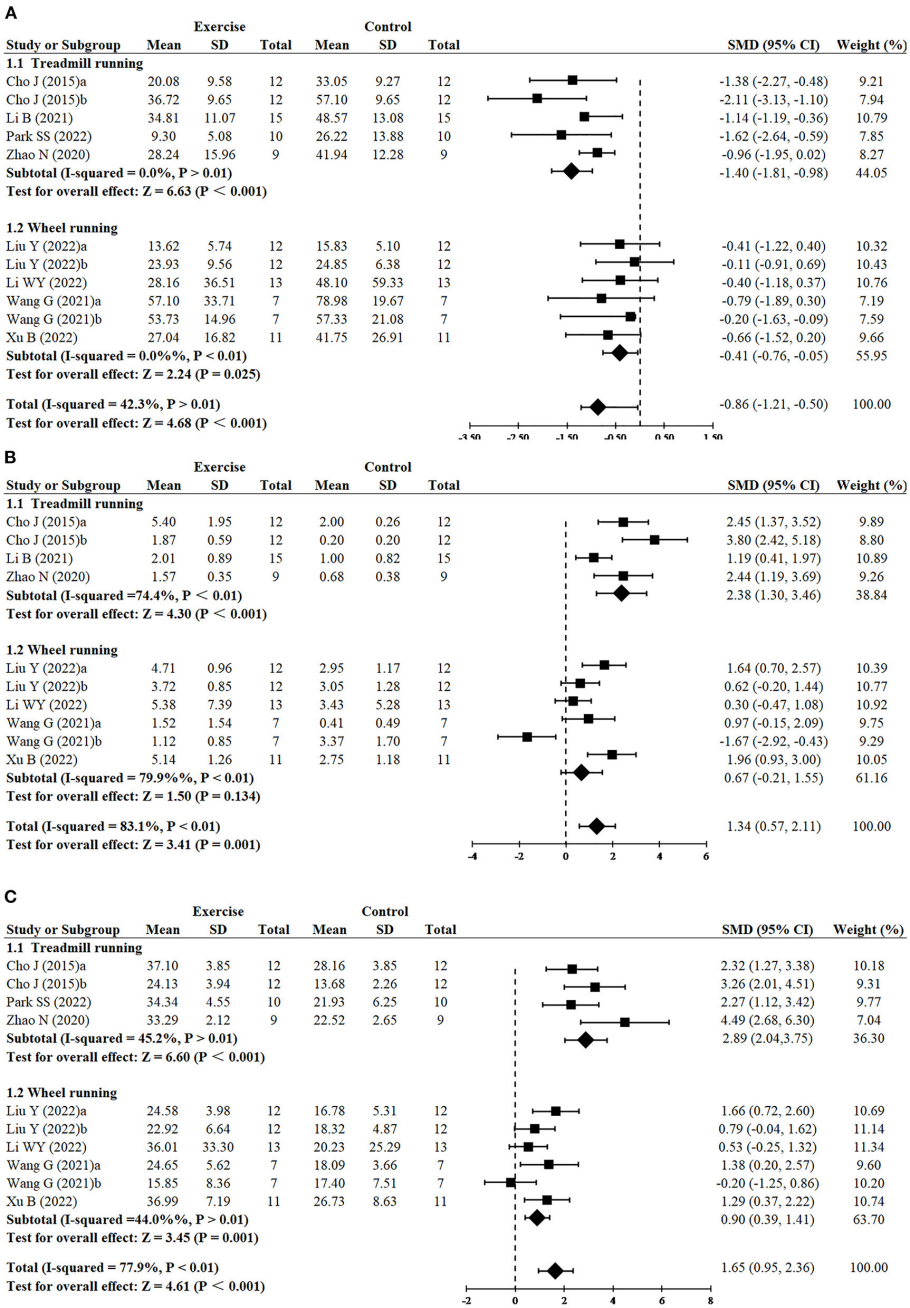
Subgroup analysis of exercise type on cognitive function in AD models. **(A)** Subgroup analysis of exercise type on the escape latency of MWM. **(B)** Subgroup analysis of exercise type on the number of platform crossings of MWM. **(C)** Subgroup analysis of exercise type on the time in the target quadrant of MWM.

Greater reduction of escape latency was observed in treadmill running (*n* = 5, SMD = −1.40, 95% CI: −1.81 to −0.98, *P* < 0.001) in comparison to wheel running (*n* = 6, SMD = −0.41, 95% CI: −0.76 to −0.05, *P* = 0.025) (Figure 4A). Treadmill running showed a significant effect on increasing platform crossover numbers (*n* = 4, SMD = 2.38, 95% CI: 1.30–3.46, *P* < 0.001), whereas wheel running had no significant difference (*n* = 6, SMD = 0.67, 95% CI: −0.21 to 1.55, *P* = 0.134) (Figure 4B). Greater increase of time in the target quadrant was observed in treadmill running (*n* = 4, SMD = 2.89, 95% CI: −2.04 to 3.75, *P* < 0.001), compared with wheel running (*n* = 6, SMD = 0.90, 95% CI: 0.39–1.41, *P* = 0.001) (Figure 4C).

#### 3.3.6. Effect of exercise type on synaptic plasticity in AD models

**Figure 5:**
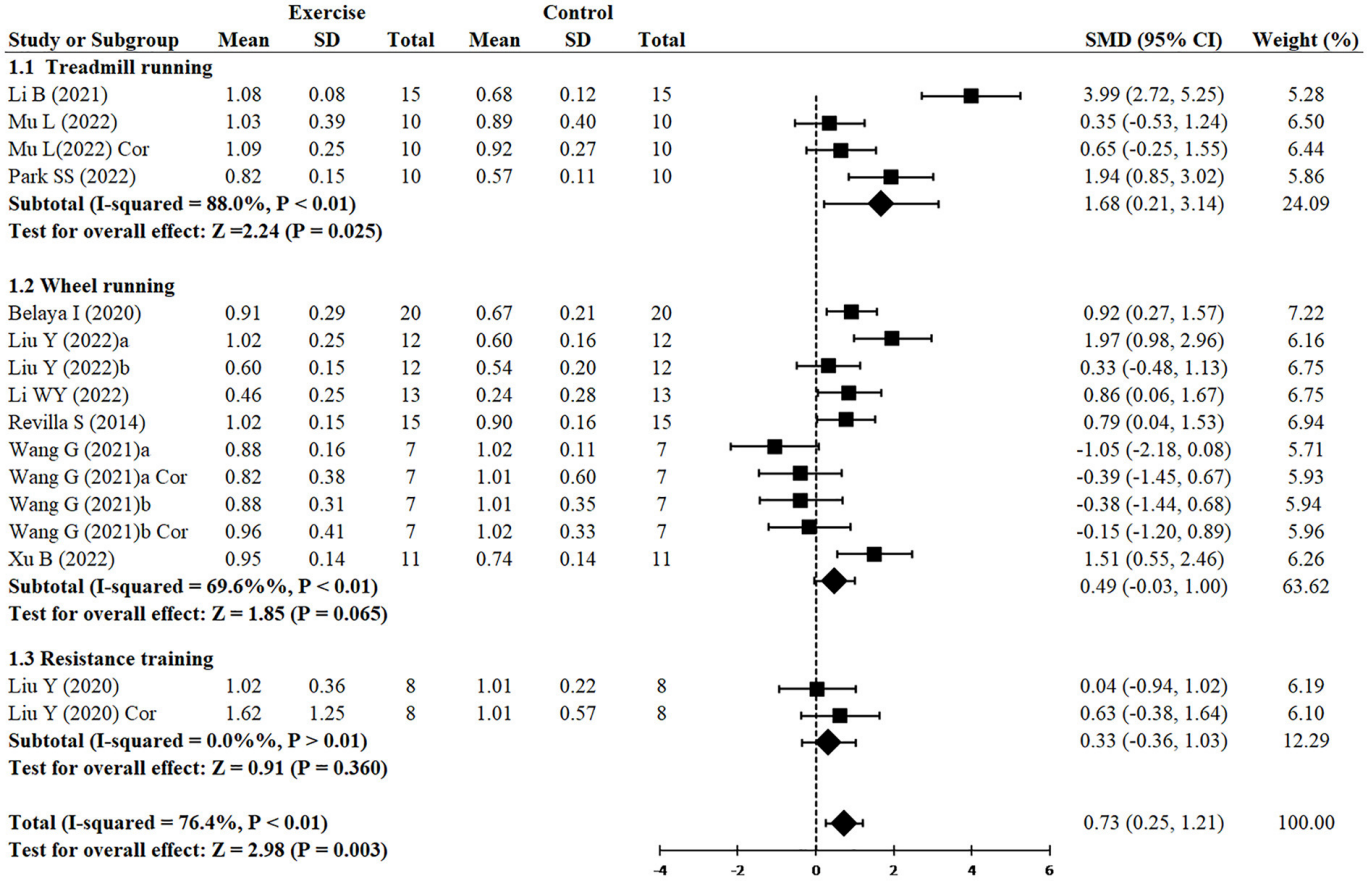
Subgroup analysis of exercise type on the expression of PSD95 in AD models.

Figure 5 demonstrated that, independently of the type of exercise, the expression of PSD95 was increased (SMD = 0.73, 95% CI: 0.25–1.21, *P* = 0.003). However, treadmill running demonstrated greater increase of PSD95 (*n* = 4, SMD = 1.68, 95% CI: 0.21–3.14, *P* = 0.025), whereas wheel running (*n* = 10, SMD = 0.49, 95% CI: −0.03 to 1.00, *P* = 0.065) and resistance training (*n* = 2, SMD = 0.33, 95% CI: −0.37 to 1.03, *P* = 0.360) had no significant difference.

### 3.4. Results of the qualitative analysis

#### 3.4.1. Effect of exercise on synaptic plasticity in AD models: Dendritic spine density

Totally, three studies (Mu et al., 2022; Xu et al., 2022; Yang et al., 2022) have reported an association between a higher density of dendritic spines and exercise. Dendritic spines are small projections on the dendrite stem that form synapses with the axons of neurons to receive and integrate information (Chidambaram et al., 2019), which are highly correlated with hippocampus-dependent spatial navigation (Bolding et al., 2019). The results displayed that the dendritic spine density of the hippocampus was obviously higher in the exercise group than the sedentary controls. Furthermore, two studies (Mu et al., 2022; Xu et al., 2022) reported in detail that exercise pretreatment blocked the decrease in the number of thin spines, mushroom spines, and stubby spines both in the hippocampus and prefrontal cortex. In the study by (Mu et al., 2022), there was also reported that treadmill exercise enhanced the axon length and dendritic complexity.

#### 3.4.2. Effect of exercise on synaptic plasticity in AD models: The number of synapses

Overall, three articles (Li et al., 2021; Mu et al., 2022; Yang et al., 2022) mentioned a positive correlation between more synapses and exercise. And two articles (Li et al., 2021; Mu et al., 2022) further measured and analyzed the ultra-structural parameters by electron microscopy. They indicated that exercise also increased the length of the synaptic active zone, the width of the synaptic cleft, synaptic curvature, and the thickness of the postsynaptic density in the hippocampus, which greatly improved the efficiency of synaptic transmission. In addition to the hippocampus, Mu et al. (2022) and Yang et al. (2022) also evaluated the positive effect of exercise on the prefrontal cortex.

#### 3.4.3. Effect of exercise on synaptic plasticity in AD models: LTP

In all, four studies (Dao et al., 2015, 2016; Zhao et al., 2015; Lourenco et al., 2019) used electrophysiological experiments to detect LTP, which found that PS amplitude and fEPSP slope were reduced significantly in AD models. LTP is a form of synaptic plasticity accepted as an electrophysiological model of learning and memory (Zhao et al., 2015). All results showed that exercise could increase the fEPSP slope significantly in both young and old AD models, indicating that exercise prevented the impairment of synaptic transmission. Nevertheless, one study (Dao et al., 2016) reported that exercise only partially prevented the effect of Aβ on PS amplitude. Different from the aforementioned studies, Dao et al. (2015) and Zhao et al. (2015) reported that there was no significant difference of PS amplitude between the sedentary and exercise mice.

### 3.5. Sensitivity analysis

We performed sensitivity analysis based on the outcomes of escape latency, number of platform crossings, time in the target quadrant, and the expression of PSD95. After each study was successively removed, the effects of the remaining studies were within the 95% CI of the total effect. It demonstrated that the meta-analysis had a low level of sensitivity and produced stable and reliable results (Supplementary Figures 1–4).

### 3.6. Publication bias

**Figure 6:**
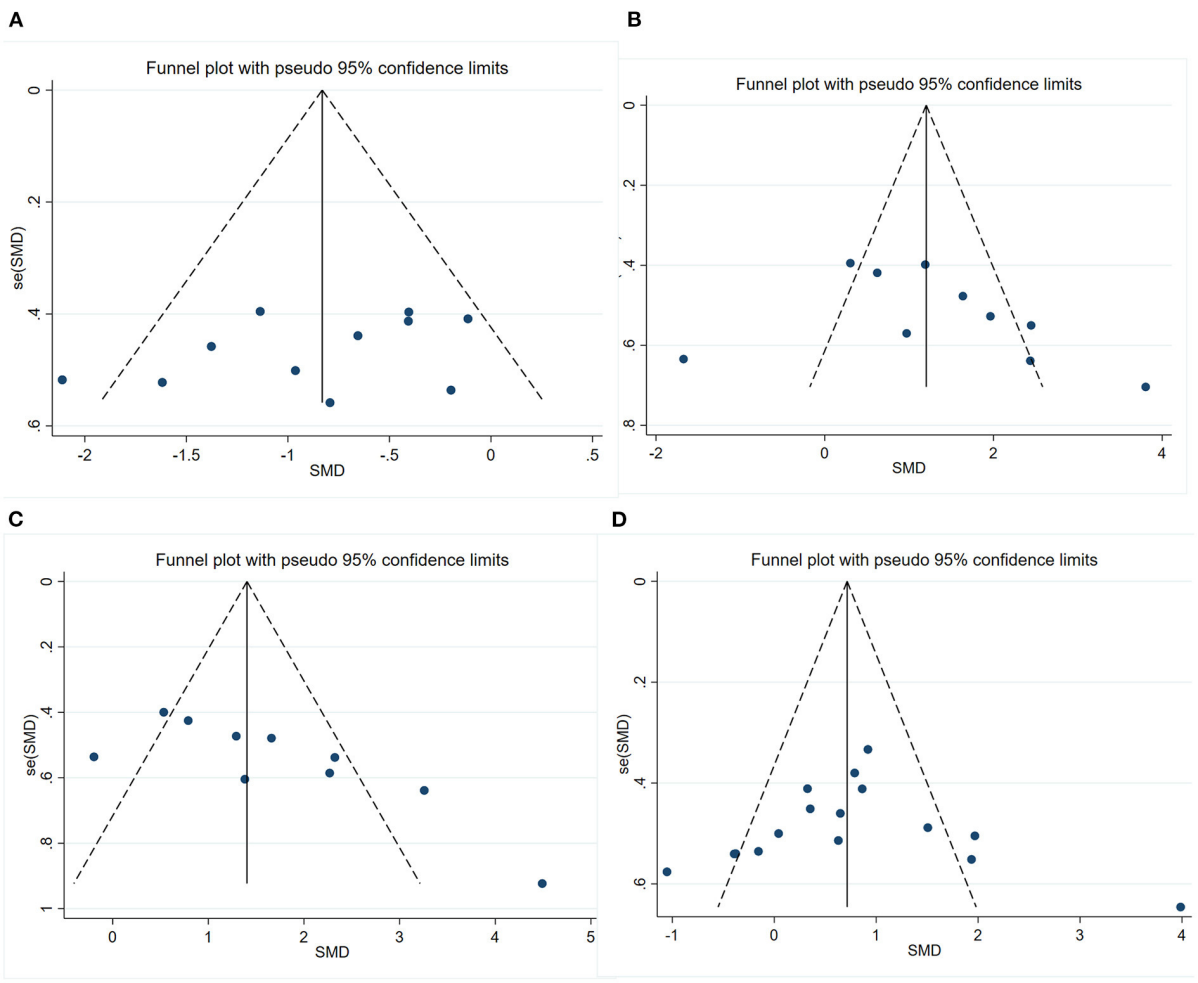
Funnel plots showing the effect of exercise on cognitive function and synaptic plasticity in AD models. **(A)** Funnel plot for the effect of exercise on the escape latency of MWM. **(B)** Funnel plot for the effect of exercise on the number of platform crossings of MWM. **(C)** Funnel plot for the effect of exercise on the time in the target quadrant of MWM. **(D)** Funnel plot for the effect of exercise on the expression of PSD95.

We conducted publication bias test with funnel plot (Figure 6) and Egger's test for the four outcomes. The symmetry indicating there was no significant publication bias of escape latency (*P* = 0.211) and number of platform crossings (*P* = 0.310). However, the funnel plot's visual inspection revealed significant asymmetry and the Egger's test identified potential evidence of publication bias of time in the target quadrant (*P* = 0.012) and PSD95 (*P* < 0.001). Then, a sensitivity analysis using the trim-and-fill method was performed, which indicated that there was no need to trim any existing studies and fill any other unpublished studies. Therefore, it was considered to have no significant risk of publication bias and the risk of bias caused by other factors.

## 4. Discussion

To our knowledge, this is the first systematic review and meta-analysis to examine the effect of exercise on cognitive and synaptic function in animal models of AD, using the MWM test and synaptic plasticity as outcome indicators. Our study highlighted the positive effect of exercise on cognitive function and synaptic plasticity in animal models of AD. And the effect of exercise on cognitive function and synaptic plasticity was more significant in the exercise before AD group than the exercise after AD group. Our findings suggested that exercise had a potential effect on improving cognitive and synaptic function in animal models of AD.

According to reports from four included studies (Dao et al., 2015, 2016; Zhao et al., 2015; Lourenco et al., 2019), exercise can improve LTP damage by increasing the slope of field excitatory postsynaptic potential (fEPSP). It was consistent with Loprinzi PD's conclusions (Moore and Loprinzi, 2021). This may be related to the fact that exercise stimulated skeletal muscles. According to studies, active skeletal muscles can secrete numerous substances that have a protective effect on the brain (Sui et al., 2021). Western blotting and *in vivo* electrophysiology had indicated that exercise could increase Calcium/calmodulin-dependent protein kinase IV (CaMKIV) availability, which in turn will enhance cAMP-response element binding protein (CREB) phosphorylation and subsequent CREB-mediated transcriptions (Dao et al., 2016). CREB regulates the transcription of brain-derived neurotrophic factor (BDNF), which is closely related to LTP and spatial long-term memory (Brann et al., 2021). Moreover, analysis of the number of synapses and spine density in five included studies (Kim et al., 2014; Li et al., 2021; Mu et al., 2022; Xu et al., 2022; Yang et al., 2022) showed that exercise increased the number of synapses and spines, especially the thin and mushroom-type spines in the hippocampus of AD. BDNF played an essential role in this process (von Bohlen Und Halbach and von Bohlen Und Halbach, 2018). Furthermore, both BDNF signaling and its downstream mediators CREB and CaMKIV could be regulated by uncoupling protein 2 (UCP2) (Dao et al., 2015). Based on these results, it is possible to hypothesize that exercise may modulate synaptic plasticity by affecting UCP2. Exercise probably increases fEPSP by regulating UCP2, thus ameliorating LTP impairment. However, the mechanism is still unclear, and more research is needed to explore and clarify the mechanism in the future.

PSD95 is a key synaptic protein that regulates synaptic transmission and plasticity. Our results showed that exercise increased the expression of PSD95 in the brain of AD models (SMD = 0.73, *P* = 0.003). There was evidence indicating that the expression of PSD95 was regulated by Neuregulinl (NRG1), which is a signaling protein that mediates interactions between cells and is essential for the development and maintenance of the nervous system (Ting et al., 2011). NRG-1-ICD, a hydrolytic product of NRG-1, entered the nucleus and interacted with transcription factors of zinc finger structure (Sun et al., 2019; Padjasek et al., 2020), eventually upregulating the transcriptional activity of the PSD95 promoter and increasing the expression of PSD95. Besides, it has been shown that the NRG1 signaling pathway can be regulated through exercise (Ennequin et al., 2015). Therefore, we hypothesize that exercise probably increases PSD95 expression by activating the NRG1 signaling pathway and promoting the movement of NRG-1-ICD into the nucleus. However, it requires more future exploration.

Our subgroup analysis found that exercise before AD had a superior effect on cognitive function and synaptic improvement than exercise after AD. Particularly for the number of platform crossings (1.71 vs. 0.73 SMD for exercise before AD and exercise after AD, respectively) and the expression of PSD95 (0.94 vs. 0.47 SMD for exercise before AD and exercise after AD, respectively). It may be related to the different degrees of pathological changes in animal models of AD in different cognitive status. Compared with the subgroup of exercise before AD, animals in the subgroup of exercise after AD were older. They had severe pathological changes like plaque deposition and neuroinflammation in the brain (Zhao et al., 2015), which were difficult to reverse. Thus, the effect of exercise may not be sufficient to counteract cognitive decline and AD pathological changes (Xu et al., 2013). Or there might be a minor benefit in some respects, but this does not translate into restored cognitive function after exercise. It was also confirmed by our findings, exercise had a positive effect on outcome indicators of escape latency and time in the target quadrant, but not on other indicators.

The timing of treatment was a key element in the benefits of exercise on AD (Moore and Loprinzi, 2021). Our findings were consistent with recent opinions of some scholars that prevention rather than treatment should be emphasized for AD. Therefore, if the brain function is relatively healthy, exercise can assist to prevent or delay the onset of AD. Contrarily, if nerve function is severely impaired, exercise does not prevent neurodegeneration. In this sense, to reduce the risk of AD or to slow its progression, early or preventative exercise intervention is required.

This study included six AD models: four transgenic animal models and two drug-induced animal models. APP/PS1 double-transgenic mice expressed both Swedish (KM670/671NL) mutation of the APP gene and the FAD-linked PSEN1 gene without exon 9 (dE9). It is characterized by plaque deposition and neuron loss (Yokoyama et al., 2022). The course of the disease is generally manifested as cognitive behavioral changes at 3 months of age, senile plaques at 5 months of age, and a large number of senile plaques at 12 months of age (Zhu et al., 2017). 3×Tg mice, a representative missense mutation linked to tauopathies mode mice, is characterized by plaques and neurotangles in brain regions (Yokoyama et al., 2022). Long-term memory impairment begins at 4 months of age in 3×Tg mice (Javonillo et al., 2022), which is often used to study the interactions between amyloid and tau pathologies. These two types of mice were the most used AD models in this study. 5×FAD mice were generated by coexpressing the five FAD mutations in APP/PS1 double-transgenic mice, which caused the appearance of Aβ plaques in the brain at the age of 2 months and reached saturation at 6 months, together with robust gliosis (Gatt et al., 2019; Yokoyama et al., 2022). TgF344 rats, transgenic rats bearing mutant human APP and PS1, were first reported by Robert in 2013 (Cohen et al., 2013). It manifests age-dependent cerebral amyloidosis, tauopathy, gliosis, apoptotic loss of neurons, and cognitive disturbance. Injection of Aβ and streptozotocin is relatively simple, reproducible, and stable. However, drug induction only targets a certain aspect of AD pathology, which is far from the complicated pathological process of AD (Kamat, 2015). It should be noted that the phenotypes of different AD models were different, however, they gradually captured the full pathology of AD with aging, including cognitive impairment and synaptic damage. As described above, there were various types of AD models, which played an important role in research, especially in AD research.

In our review, eight included studies (Cho et al., 2015; Zhao et al., 2020; Li et al., 2021, 2022; Wang et al., 2021; Liu et al., 2022; Park et al., 2022; Xu et al., 2022) assessed hippocampal-dependent learning and memory using MWM test. We found that exercise could reduce the time of escape latency (SMD = −0.86, *P* < 0.001), increase crossovers (SMD = 1.34, *P* = 0.001) and time in the target quadrant (SMD = 1.65. *P* < 0.001) in animal models of AD. However, when using a large pool diameter or shrinking the platform-site size, platform crossings measured during the trials may be biased (Othman et al., 2022). The pool diameters of the included studies used were 100 cm (Liu et al., 2022), 120 cm (Cho et al., 2015; Li et al., 2022; Xu et al., 2022) and 150 cm (Zhao et al., 2020) and platform diameters were 9 cm (Xu et al., 2022), 10 cm (Li et al., 2022) and 12 cm (Zhao et al., 2020), respectively. Furthermore, only three studies performed adaptation training to detect complete visual function in mice (Zhao et al., 2020; Li et al., 2022; Park et al., 2022). Other studies did not report these details, which may have biased the results somewhat (Vorhees and Williams, 2014). Hence, experimental tools should be carefully selected and report these details as much as possible in future trials.

There were several limitations to our study. Firstly, the included studies used multiple AD models, and the size of the sample group was relatively small, which may influence the effects of exercise. But the number of included studies was relatively small and did not allow for subgroup analysis according to types of AD models. Secondly, the studies included did not have data on the senescence-accelerated mouse prone-8 (SAMP8) mice, a non-transgenic but robust AD model, which played an important role in anti-aging research. Thirdly, the MWM tools used in the included studies were inconsistent. A platform suitable for the size of the experimental animal should be selected to obtain more rigorous results. Finally, the quality of included studies was not ideal, and some details were poorly reported or even absent, such as unclear random allocation methods and the lack of randomization and blinding principles. Based on the above limitations, future studies should be rigorously designed, and the results should be reported completely and transparently.

## 5. Conclusion

In conclusion, our review provided evidence that exercise had a positive effect on improving learning memory ability and enhancing synaptic plasticity of AD. The results of the subgroup analysis also indicated that early or preventive exercise intervention is a better way to reduce the risk or slow the progression of AD. Further investigations on the mechanisms underlying the beneficial effects of exercise on AD are warranted. However, there was a certain heterogeneity in the included studies, and results should be reported with caution.

## Data availability statement

The original contributions presented in the study are included in the article/Supplementary material, further inquiries can be directed to the corresponding author/s.

## Author contributions

LG and YL contributed to the subject design and conception of this review. LG, XY, and YZ contributed to retrieving materials and extracting data, summarized the extracted data, and performed the data analysis. LG, XY, and XX drafted the manuscript. YL revised important parts of the manuscript. All authors reviewed and approved the final version of the manuscript.
