# Supplementary material for: Effect of exercise on cognitive function and synaptic plasticity in Alzheimer's disease models: A systematic review and meta-analysis
Source: Front Aging Neurosci. 2023 Jan 10;14:1077732. doi: 10.3389/fnagi.2022.1077732 (PMC9872519; doi:10.3389/fnagi.2022.1077732)
Supplement: Supplementary file 1 [file Data_Sheet_1.zip › Search strategy.DOCX]

Supplementary Material

Search strategies

855 of PubMed

| Search number | Query | Results |
| --- | --- | --- |
| #1 | （Exercise [MeSH Terms] ）OR (Resistance Training [MeSH Terms]) OR (physical exercise) OR (aerobic) OR (treadmill) OR (running) OR (voluntary) OR (involuntary) OR (swimming) | 473199 |
| #2 | (Synapses [MeSH Terms]) OR (Neuronal Plasticity [MeSH Terms]) OR (synaptic) OR (neuroplasticity) OR (plasticity) OR (synaptogenesis) OR (dendritic) OR (dendron) OR (long term potentiation) OR (LTP) | 362659 |
| #3 | (Alzheimer Disease [MeSH Terms]) OR (Dementia [MeSH Terms]) OR (Cognition [MeSH Terms]) OR (Alzheimer) OR (AD) OR (cognitive) | 794252 |
| #4 | #1 AND #2 AND #3 | 1333 |
| #5 | #1 AND #2 AND #3 AND (2012/4/1:2022/4/1[pdat]) | 855 |

2502 of Embase

| NO. | Query2502 of Embase | Results |
| --- | --- | --- |
| #1 | 'exercise'/exp OR 'resistance training'/exp OR 'physical exercise' OR 'aerobic' OR 'treadmill' OR 'running' OR 'voluntary' OR 'involuntary' OR 'swimming' | 710476 |
| #2 | 'synapse'/exp OR 'nerve cell plasticity'/exp OR 'synaptic' OR 'neuroplasticity' OR 'plasticity' OR 'synaptogenesis' OR 'dendritic' OR 'dendron' OR 'long-term potentiation' OR 'ltp' | 453108 |
| #3 | 'alzheimer disease'/exp OR 'dementia'/exp OR 'cognition'/exp OR 'alzheimer' OR 'ad' OR 'cognitive' | 3398422 |
| #4 | #1 AND #2 AND #3 | 3408 |
| #5 | #1 AND #2 AND #3 AND [2012-2022]/py | 2502 |

1312 of Medline

| ID | Search | Results |
| --- | --- | --- |
| #1 | exercise OR resistance training OR physical exercise OR aerobic OR treadmill OR running OR voluntary OR involuntary OR swimming | [812684](http://apps.webofknowledge.com/summary.do?product=UA&doc=1&qid=959&SID=6COXGwZSwWxP9dHFJu3&search_mode=GeneralSearch&update_back2search_link_param=yes) |
| #2 | synapse* OR neuronal plasticity OR synaptic OR neuroplasticity OR plasticity OR synaptogenesis OR dendritic OR dendron OR long-term potentiation OR LTP | 396525 |
| #3 | Alzheimer disease OR dementia OR cognition OR Alzheimer OR AD OR cognitive | [793561](http://apps.webofknowledge.com/summary.do?product=UA&doc=1&qid=964&SID=6COXGwZSwWxP9dHFJu3&search_mode=GeneralSearch&update_back2search_link_param=yes) |
| #4 | #1 AND #2 AND #3 | [1678](http://apps.webofknowledge.com/summary.do?product=UA&doc=1&qid=965&SID=6COXGwZSwWxP9dHFJu3&search_mode=GeneralSearch&update_back2search_link_param=yes) |
| #5 | #1 AND #2 AND #3 AND 2012-04-01 to 2022-04-01 (Publication Date) | 1312 |

2921 of Web of science

| ID | Search | Results |
| --- | --- | --- |
| #1 | exercise OR resistance training OR physical exercise OR aerobic OR treadmill OR running OR voluntary OR involuntary OR swimming | [1927411](http://apps.webofknowledge.com/summary.do?product=UA&doc=1&qid=959&SID=6COXGwZSwWxP9dHFJu3&search_mode=GeneralSearch&update_back2search_link_param=yes) |
| #2 | synapse* OR neuronal plasticity OR synaptic OR neuroplasticity OR plasticity OR synaptogenesis OR dendritic OR dendron OR long-term potentiation OR LTP | 1001774 |
| #3 | Alzheimer disease OR dementia OR cognition OR Alzheimer OR AD OR cognitive | [1445320](http://apps.webofknowledge.com/summary.do?product=UA&doc=1&qid=964&SID=6COXGwZSwWxP9dHFJu3&search_mode=GeneralSearch&update_back2search_link_param=yes) |
| #4 | #1 AND #2 AND #3 | [3840](http://apps.webofknowledge.com/summary.do?product=UA&doc=1&qid=965&SID=6COXGwZSwWxP9dHFJu3&search_mode=GeneralSearch&update_back2search_link_param=yes) |
| #5 | #1 AND #2 AND #3 AND 2012-04-01 to 2022-04-01 (Publication Date) | 2921 |

836 of Cochrane Library

| ID | Search | Hits |
| --- | --- | --- |
| #1 | exercise OR resistance training OR physical exercise OR aerobic OR treadmill OR running OR voluntary OR involuntary OR swimming | 151055 |
| #2 | synapses OR neuronal plasticity OR synaptic OR neuroplasticity OR plasticity OR synaptogenesis OR dendritic OR dendron OR long-term potentiation OR LTP | 24468 |
| #3 | Alzheimer disease OR dementia OR cognition OR Alzheimer OR AD OR cognitive | 156720 |
| #4 | #1 AND #2 AND #3 with Cochrane Library publication date Between Apr 2012 and Apr 2022 | 836 |
