# Supplementary material for: Effect of exercise on cognitive function and synaptic plasticity in Alzheimer's disease models: A systematic review and meta-analysis
Source: Front Aging Neurosci. 2023 Jan 10;14:1077732. doi: 10.3389/fnagi.2022.1077732 (PMC9872519; doi:10.3389/fnagi.2022.1077732)
Supplement: Supplementary file 1 [file Data_Sheet_1.zip › Supplementary Figure 3.DOCX]

Supplementary Material

## Supplementary Figures


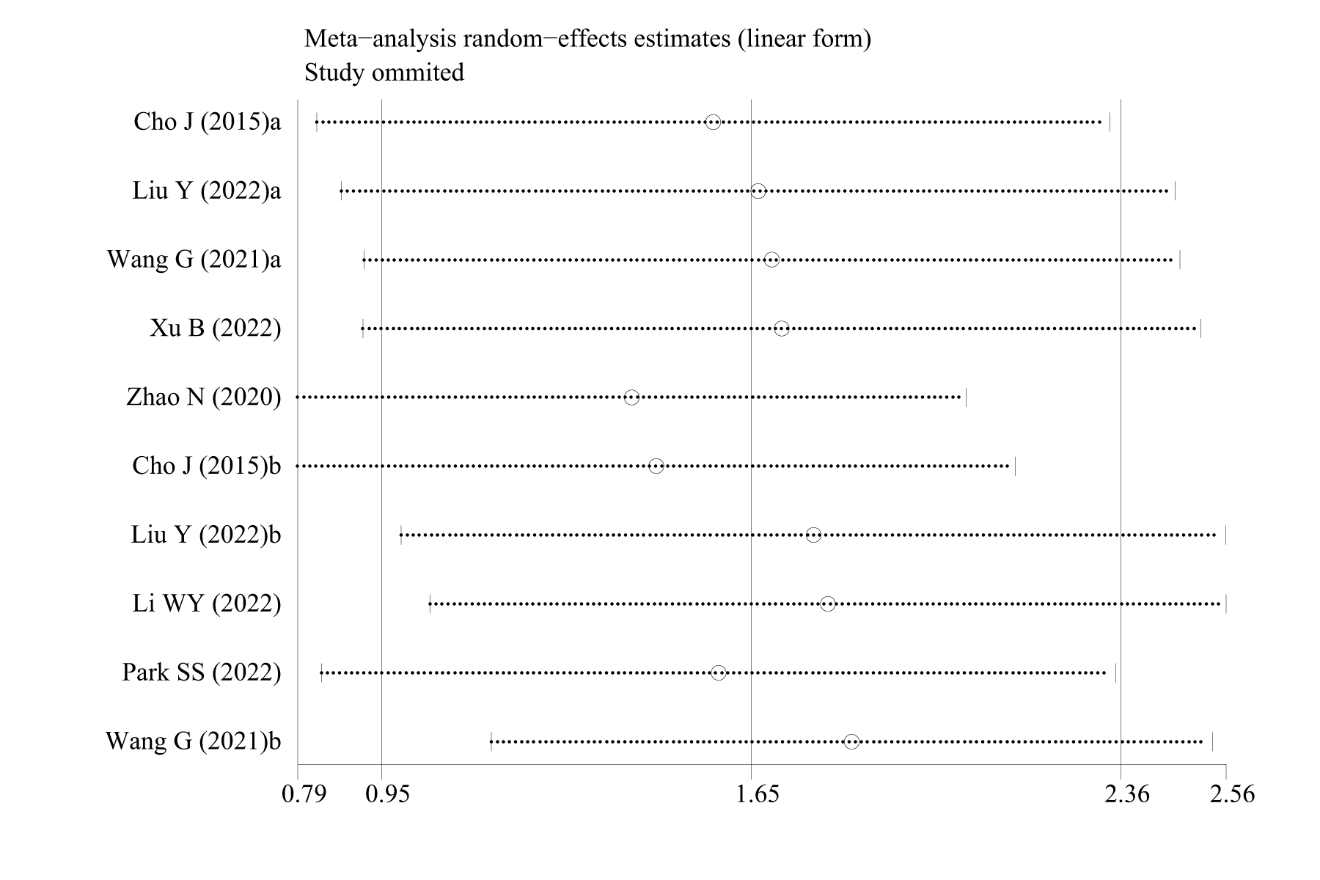


**Supplementary Figure 3.** Sensitivity analysis result of exercise on the time in the target quadrant in each study.
